# Type of Multimorbidity and Patient-Doctor Communication and Trust among Elderly Medicare Beneficiaries

**DOI:** 10.1155/2016/8747891

**Published:** 2016-10-05

**Authors:** Rahul Garg, Chan Shen, Nethra Sambamoorthi, Kimberly Kelly, Usha Sambamoorthi

**Affiliations:** ^1^Department of Pharmaceutical Systems and Policy, West Virginia University, Morgantown, WV 26506, USA; ^2^Department of Biostatistics and Health Services Research, University of Texas MD Anderson Cancer Center, Houston, TX 77030, USA; ^3^School of Continuing Education, Northwestern University, Evanston, IL 60208, USA

## Abstract

*Background*. Effective communication and high trust with doctor are important to reduce the burden of multimorbidity in the rapidly aging population of the US. However, the association of multimorbidity with patient-doctor communication and trust is unknown.* Objective*. We examined the relationship between multimorbidity and patient-doctor communication and trust among the elderly.* Method*. We used the Medicare Current Beneficiary Survey (2012) to analyze the association between multimorbidity and patient-doctor communication and trust with multivariable logistic regressions that controlled for patient's sociodemographic characteristics, health status, and satisfaction with care.* Results*. Most elderly beneficiaries reported effective communication (87.5–97.5%) and high trust (95.4–99.1%) with their doctors. The elderly with chronic physical and mental conditions were less likely than those with only physical conditions to report effective communication with their doctor (Adjusted Odds Ratio [95% Confidence Interval] = 0.80 [0.68, 0.96]). Multimorbidity did not have a significant association with patient-doctor trust.* Conclusions*. Elderly beneficiaries had high trust in their doctors, which was not affected by the presence of multimorbidity. Elderly individuals who had a mental condition in addition to physical conditions were more likely to report ineffective communication. Programs to improve patient-doctor communication with patients having cooccurring chronic physical and mental health conditions may be needed.

## 1. Introduction

Patient-doctor relationship has been defined as a meaningful therapeutic interaction between the patients and their doctors and is considered as the seventh element of healthcare quality [1]. Meaningful therapeutic interaction between patients and doctors includes both patient-doctor communication and trust [2]. An effective patient-doctor communication consists of explaining patient's illnesses and treatments, spending adequate time, managing uncertainty, and developing rapport [3–5]. Patient-doctor trust is high when the doctor is technically competent, is thorough and careful in examining the patient, has good listening capabilities, and has complete understanding of patient's illnesses and treatments [6–10].

Effective communication has been found to enhance patient's engagement in care [11], satisfaction with care [12], and treatment adherence [13]. Effective communication has also been found to improve health outcomes [14, 15]. Among older patients with diabetes, patient-doctor communication was associated with better diabetes self-management [14]. Patient-doctor communication has been found to be associated with lower blood pressure, lower anxiety, and better quality of life [15]. Similarly, high trust in the doctor has been found to be associated with lower hassles in self-care, better ability to perform diabetes care activities [16], and satisfaction with the doctor [17].

Effective patient-doctor communication and trust become especially important in managing patients with two or more chronic conditions (i.e., multimorbidity). Multimorbidity affects elderly population disproportionately and is projected to increase from 34,835 million in 2000 to 81,999 million by 2050 [18]. Multimorbidity has been reported to have adverse health consequences. Elderly individuals with multimorbidity account for 98% of hospital readmissions, incur 93% of total Medicare spending [19], and experience higher rates of preventable hospitalizations as compared to those without multimorbidity [20]. Multimorbidity also affects care coordination [21] and leads to poor management of cooccurring conditions due to the focus on one dominant chronic condition [22]. Therefore, policymakers, providers, and researchers have emphasized the need for meaningful interactions between patients and doctors in managing the burden of multimorbidity [23–26]. An effective communication and high trust with the doctor can improve the care of the elderly with multimorbidity.

A special case of multimorbidity arises when an individual has coexisting chronic physical and mental health conditions. Presence of mental health condition in addition to chronic physical conditions can be very challenging for disease management [27–30]. Effective communication and trust have been shown to improve outcomes for not only individuals with chronic physical conditions but also those with a mental health condition. For example, effective communication decreased the Patient Health Questionnaire (PHQ-9) scores among adults with depression from baseline to one-month follow-up, indicating an improvement in the severity of depression [31]. However, a survey sponsored by The National Depressive and Manic-Depressive Association (DMDA) found that ineffective communication exists between primary care physicians and patients with depression [32]. For example, the survey found that even though doctors thought that they explained the potential adverse effects of antidepressant medications, the patients felt a lack of information on certain adverse effects such as sexual dysfunction, weight gain, and insomnia [32]. Also, trust in the doctor is important for patients with mental illness to maintain the confidentiality of their mental illness due to its associated social and personal stigma [33].

There is a lack of research on the impact of having a mental health condition in addition to multiple chronic physical illnesses on communication and trust with the doctor among elderly individuals [34]. We could only find two studies (one in the US and one in Germany), which evaluated the relationship between number of chronic conditions and patient-doctor relationship [35, 36]. In the US, a study among noninstitutionalized adults aged 18 years or older from 12 metropolitan communities analyzed the relationship between number of chronic conditions and scores on patient-doctor communication scale [35]. The study found a small but significant relationship between higher number of chronic conditions and ineffective patient-doctor communication [35]. Another study among the German adults examined the factors associated with patient-doctor relationship which was measured using the German version of the Patient-Doctor Relationship Questionnaire (PDRQ-9) [36]. The study found that individuals with lower number of chronic physical and mental comorbidities reported higher scores on the PDRQ-9, indicating the higher quality of patient-doctor relationship.

Therefore, the primary objective of this study is to examine patient-doctor communication and trust among the elderly who had both chronic mental and physical conditions as compared to those having multiple physical conditions without any mental condition. We hypothesized that elderly patients having both mental and physical conditions will be more likely to have ineffective communication and have low trust with the doctor as compared to those having multiple physical conditions without any mental health condition.

## 2. Conceptual Framework

According to the previous studies, factors other than multimorbidity can affect meaningful therapeutic interactions between the patients and their doctors. These may include age, gender, race/ethnicity [37, 38], socioeconomic status, health status [39], and length of the visit [40]. Therefore, we utilized the theoretical model of patient-doctor relationship by Gabay (2015) to guide the selection of independent variables used in our study [41]. According to this model, patient-doctor relationship is influenced by (a) sociodemographic factors such as patient's age, race/ethnicity, gender, education, insurance, and metropolitan area; (b) health condition factors including multimorbidity and body mass index; (c) health status such as perceived general health status and functional health status; and (d) patient's satisfaction with care.

## 3. Methods

### 3.1. Study Design

We adopted a cross-sectional study design using data from a nationally representative survey of Medicare beneficiaries.

### 3.2. Data Source

We utilized data from the access to care module of the Medicare Current Beneficiary Survey (MCBS) 2012. The MCBS is a continuous multipurpose survey of a nationally representative sample of Medicare beneficiaries conducted by the Centers for Medicare & Medicaid Services [42, 43]. The MCBS is conducted among all Medicare beneficiaries who are above 65 years of age or who are disabled [44]. The survey is conducted through Computer-Assisted Personal Interviews (CAPI) with the beneficiaries. The person level survey data is released in two modules: access to care (AC) and Cost and Use (CU). We utilized the AC module of the MCBS that uses valid and reliable survey questions to assess patients' experiences with care received from their provider. The AC module represents the population of individuals who were always enrolled in the Medicare during the calendar year. It contains deidentified information on each participant's access to health care, usual source of care, and satisfaction with care. The AC module also includes demographic, health insurance, health status, and functioning data.

The MCBS is a longitudinal panel survey, where individuals are interviewed three times a year for a maximum of four years. The participants are selected from the Medicare enrollment file using a multistage and stratified sampling design to be representative of the Medicare population [44]. The multistage and stratified sampling design is commonly used in large national surveys to feasibly conduct survey, reduce bias, and produce nationally representative survey results. In the multistage design, whole population is divided into groups or clusters. Then, the sampling units within the clusters are chosen randomly. It avoids the sampling of all the units which can be expensive and time consuming. In stratified sampling design, the beneficiaries are divided into seven strata based on age: 0 to 44, 45 to 64, 65 to 69, 70 to 74, 75 to 79, 80 to 84, and 85 or older. The sampling units are then selected from each stratum so that the results can be representative of the total population.

### 3.3. Study Sample

**Figure 1:**
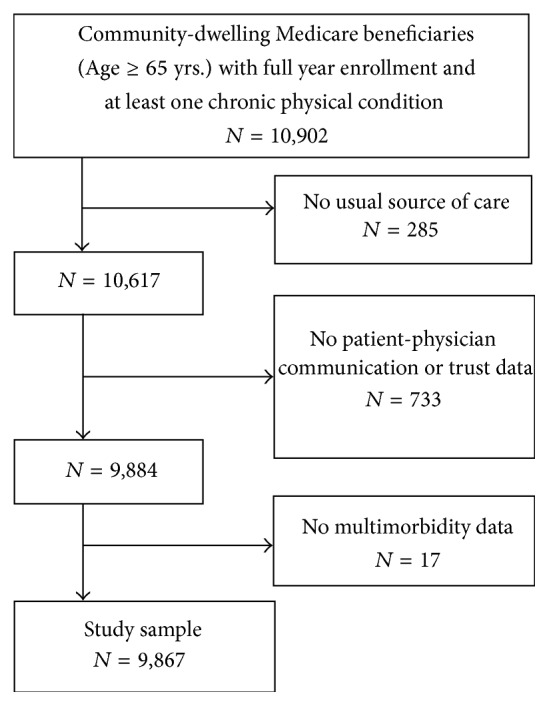
Flowchart of study sample.

We included community-dwelling elderly Medicare beneficiaries who were aged 65 years or older, had at least one chronic physical condition, and had a usual source of care in our study. Individuals were classified as having a usual source of care if they answered “yes” to the question “Is there a particular medical person or a clinic you usually go to when you are sick or for advice about your health?” Further, we excluded the individuals with missing data for chronic health conditions, communication, or trust variables. The final study sample included 9,867 elderly Medicare beneficiaries (Figure 1).

### 3.4. Measures

We utilized the participants' responses to the survey questions from 2012 AC module of the MCBS in this study. A survey item in the MCBS asked about the specialty of participants' doctor. About 90% of beneficiaries answered “family practice,” “general practice,” or “internal medicine.” Thus, most beneficiaries referred to their primary care physician while responding to the survey items about communication and trust with their doctor.

#### 3.4.1. Dependent Variable: Patient-Doctor Communication

An effective patient-doctor communication consists of discussing patient's illness and treatment, spending adequate time, building rapport, and managing patient's uncertainty [3, 5]. We used the following six survey items from the MCBS which assessed these components of communication with the doctor to construct a patient-doctor communication scale (Table 1): (a) Your doctor tells you all you want to know about your condition and treatment, (b) Your doctor often does not explain your medical problems to you, (c) You often have health problems that should be discussed but are not, (d) Your doctor often seems to be in a hurry, (e) Your doctor often acts as though he was doing you a favor by talking to you, and (f) Your doctor answers all questions. The response scale ranged from one to four (1: strongly agree, 2: agree, 3: disagree, and 4: strongly disagree). We reverse coded the survey items (a) and (f) to make their scores consistent with other items. The sum of scores of these six survey items ranged from 6 to 24 and was highly skewed. Thus, we dichotomized the total score on communication scale by using median split method to create a binary response variable [45]. The elderly with total communication score equal to or above the median value (18) were categorized as having effective communication with the doctor while those with scores below median value were considered to experience ineffective communication.

#### 3.4.2. Dependent Variable: Patient-Doctor Trust

Patient's trust in the doctor is influenced by technical skills of the doctor, thorough examination of the patient, and complete understanding of patient's medical conditions [6–10]. We utilized the following five survey items from the MCBS which measured these components of trust to construct patient-doctor trust scale: (a) Your doctor is very careful to check everything when examining you, (b) Your doctor is competent and well-trained, (c) Your doctor has a good understanding of your medical history, (d) Your doctor has a complete understanding of the things that are wrong with you, and (e) You have great confidence in your doctor. The response scale ranged from one to four (1: strongly agree, 2: agree, 3: disagree, and 4: strongly disagree). We reverse coded these five trust items so that higher scores indicate high trust. The sum of scores of these five survey items ranged from 5 to 20 and was highly skewed. Hence, we dichotomized the total score on trust scale by using median split method to create a binary response variable [45]. The elderly with a total score equal to or above median value (17) were categorized as having high trust in the doctor while those with scores below median value were considered to have low trust in the doctor.

#### 3.4.3. Key Independent Variable: Health Condition-Multimorbidity

We utilized participants' having been told by a doctor that they have a chronic condition to assess the presence of multimorbidity. We selected chronic conditions based on the conceptual framework by Goodman and colleagues for defining and measuring chronic conditions for research, policy, program, and practice [46]. The chronic physical conditions included arthritis (osteoarthritis or rheumatoid arthritis), cancer (skin or any other cancer), diabetes (type 1 or type 2), heart disease (angina pectoris, myocardial infarction, or any other heart condition), hypertension, osteoporosis, and respiratory diseases (asthma, chronic obstructive pulmonary disease, or emphysema). The mental health conditions consisted of depression or any other mental or psychiatric disorder. Based on the presence of these chronic physical and mental health conditions, we defined three multimorbidity categories: (a) no MM (no multimorbidity, that is, only one physical condition), (b) MM-PI (two or more physical conditions but no mental illness), and (c) MM-PI&MI (both physical and mental health conditions). We also included patient's body mass index (underweight/normal, overweight, and obese/morbid obese) as a health condition factor.

#### 3.4.4. Other Explanatory Variables

*(1) Sociodemographic Factors*. The sociodemographic factors included beneficiaries' age, sex, race/ethnicity, marital status, education level, annual personal income, supplemental insurance (Medicaid) and type of insurance (HMO/FFS), and external environmental characteristics (metro status and region of residence).

*(2) Patient's Health Status*. We measured patient's self-perceived general health status as compared to others of the same age (excellent/very good, good, or fair/poor). We also measured functional health status by examining participants' self-reported difficulty with activities of daily living (ADL) including difficulty in walking, eating, bathing, dressing, getting in or out of bed or chair, or using the toilet [47–50]. We classified participants' difficulty with ADL in three categories: (a) no ADL, (b) 1-2 ADL, and (c) 3–6 ADL.

*(3) Patient's Satisfaction with Care*. Patient's satisfaction with care constitutes an important factor which can affect patient-doctor communication and trust. We used participants' responses to the survey item “Please tell me how satisfied you have been with the overall quality of the health care you have received over the past year” to assess patient's satisfaction with care [51–54]. The elderly who answered with “very satisfied” or “satisfied” to this survey item were considered satisfied while those who answered with “dissatisfied” or “very dissatisfied” were considered dissatisfied with the quality of care.

### 3.5. Statistical Analyses

We analyzed the validity of patient-doctor communication and trust scales using factor structure by the Principal Component Analysis with Promax rotation [55] and internal consistency (a measure of reliability) by Cronbach's alpha coefficient [56]. The Principal Component Analysis yielded single factor for both patient-doctor communication and trust scales. The single factor accounted for 98% of variance for the communication scale and 100% of variance for the trust scale. The alpha coefficient value for the communication scale was 0.87 and for the trust scale was 0.92, which indicated high reliability for both the scales.

We used chi-square tests to examine the unadjusted associations of patient-doctor communication and trust with theoretically derived predictor variables (e.g., patient's sociodemographic characteristics, health status, health condition, and patient's satisfaction factors). Then, correlations were used to assess multicollinearity, resulting in the removal of patient's health status (self-perceived general health and functional status) from further analysis. Multivariable logistic regressions were conducted to examine the relationship between multimorbidity and patient-doctor relationships, after adjusting for patient's sociodemographic factors, health condition, and satisfaction with care. We used survey procedures with Statistical Analysis System (SAS) software to account for the complex survey design [57].

## 4. Results

### 4.1. Study Sample Characteristics

Among 9,867 Medicare beneficiaries included in this study (Table 1), the majority were white (78.5%), lived in a metro region (77.4%), did not have limitations in activities of daily living (66.2%), were either overweight or obese (64.6%), and were satisfied with quality of care (97.4%). Also, 56.6% elderly beneficiaries were women, 57.2% were married, 45.4% had more than high school education, 35.1% were enrolled in HMO, 12.3% had Medicaid insurance, 19.6% had fair or poor general health status, and 58.6% were past or current smokers (Table 1).

### 4.2. Study Sample Characteristics by Multimorbidity

More than two-thirds of beneficiaries had MM-PI (72.0%) and 12.1% had MM-PI&MI, constituting 84.1% of beneficiaries with either type of multimorbidity. From the unadjusted analysis, MM-PI&MI was present among a higher percentage of women than men (14.5% versus 9.0%), current smokers than nonsmokers (20.2% versus 11.3%), those aged 65–69 years than 75–79 years (14.3% versus 10.6%), those having less than high school education than more than high school education (15.2% versus 10.9%), having <$20,000 annual income than >$50,000 income (16.5% versus 8.5%), and having Medicaid insurance (20.6% versus 11.0%) (Table 1).

### 4.3. Unadjusted Analysis: Multimorbidity and Patient-Doctor Communication

An overwhelming majority of the elderly strongly agreed or agreed that their doctor “tells them all that they want to know about their condition and treatment” (95.7%) and “answers all questions” (97.5%). Also, a majority of beneficiaries strongly disagreed or disagreed that “they often have health problems that should be discussed but are not” (93.6%) and that their doctor “often seems to be in a hurry” (87.5%), “often does not explain their medical problems to them” (92.2%), and “often acts as though he was doing them a favor by talking to them” (96.0%) (Table 2). From the unadjusted analysis, a lower percentage of the elderly with MM-PI&MI (80.1%) had effective communication with the doctor as compared to those with MM-PI (84.2%) and no MM (89.7%) (Table 3).

### 4.4. Unadjusted Analysis: Multimorbidity and Patient-Doctor Trust

Most elderly Medicare beneficiaries had high trust in their doctors. A majority of beneficiaries agreed or strongly agreed that “they have great confidence in their doctor” (96.6%) and that their doctor “is competent and well-trained” (99.1%), “is very careful to check everything when examining them” (95.4%), “has a good understanding of their medical history” (97.7%), and “has a complete understanding of the things that are wrong with them” (96.6%) (Table 2). From the unadjusted analysis, there was no significant difference in trust in the doctor among the elderly with different multimorbidity categories (Table 3).

### 4.5. Adjusted Analysis: Multimorbidity and Patient-Doctor Communication

From the adjusted multivariate analysis, type of multimorbidity had a significant association with patient-doctor communication. After adjusting for patient's sociodemographic characteristics, patient's health condition, and patient's satisfaction with care, Medicare beneficiaries with MM-PI&MI were significantly less likely to have effective communication with the doctor (Adjusted Odds Ratio [95% Confidence Interval] = 0.80 [0.68, 0.96]), as compared to those with MM-PI (Table 4). Further, the elderly with no MM were significantly more likely to have effective communication with the doctor (1.48 [1.21, 1.82]), as compared to those with MM-PI. Other factors significantly associated with effective communication with the doctor were higher education and higher satisfaction with the quality of care. Further, those living in the West were less likely to have efficient communication with the doctor as compared to those living in the Northeast (Table 4).

### 4.6. Adjusted Analysis: Multimorbidity and Patient-Doctor Trust

The multimorbidity did not have a significant association with elderly patient's trust in the doctor (MM-PI&MI versus MM-PI: 0.98 [0.84, 1.15]). Other variables significantly associated with high trust in the doctor included female sex, higher education, higher personal income, and higher satisfaction with the quality of care (Table 4). The elderly above 80 years of age compared to 65–69 years, those who are African American or other race compared to white race, and those living in a metropolitan area compared to nonmetropolitan area were less likely to have high trust in the doctor (Table 4).

## 5. Discussion

With multimorbidity being a norm in the US elderly population, policy focus has recently shifted towards the better illness management of these individuals [58, 59]. Elderly patients with multimorbidity need care that is responsive to the interactions between multiple conditions and treatments and is tailored to their individual needs. An effective communication and high trust between the patient and doctor are necessary for improving the care of these patients [23]. To our knowledge, this is the first study to examine the association between multimorbidity with or without a mental condition and patient-doctor communication and trust among a nationally representative population of the elderly in the US.

Our results suggest that a majority of elderly Medicare beneficiaries experience effective communication and have high trust in their doctor. However, the elderly who had a mental health condition in addition to chronic physical conditions were less likely to have effective communication as compared to the elderly who had multiple physical conditions without any mental condition. These results are consistent with a previous study that found an ineffective communication between doctor and patient with a single chronic mental condition [32]. The elderly with a mental condition have been found to have different communication needs from individuals without any mental condition. For example, nonverbal communication activities such as gesture and facial activity are impaired among patients with a mental condition [60]. The elderly with both mental and physical chronic conditions may also have social and internal stigma due to mental condition which might affect their communication with the doctor as compared to those with multiple physical conditions without mental condition [61]. Our results suggest that clinicians need to pay additional attention for an effective communication and higher trust when treating patients with concurrent physical and mental conditions.

Nearly fifteen percent of elderly beneficiaries with both chronic physical and mental conditions agreed or strongly agreed that their doctor is often in a hurry. A study by Quirk et al. found that doctor's appearing to be in a hurry during the visit is perceived as an uncaring attitude by the patients [62]. Patients place importance on having a doctor who talks at a slow pace and allows the patient to take his/her time. It is especially critical for an elderly patient with multiple chronic conditions because elderly patients and their families have to take care of all the cooccurring physical and mental health conditions at the same time and may want to talk about all of their diagnoses and comprehend numerous prescribed drugs and treatments. It is important for the clinicians not to hurry during consultation with an elderly patient having multimorbidity and allow the patient to take his/her own time to understand their multiple diagnoses and treatments.

Multimorbidity presents a challenge to primary care providers for addressing the competing demands of multiple physical and mental conditions in busy outpatient settings [63]. Discussion of multiple illnesses and treatments within a short visit may leave some patients feeling that the doctor did not discuss or provide all the information regarding their conditions and treatments, which we observed in our study. Physicians who are treating patients with chronic physical and mental conditions may want to explore some alternative solutions to improve the discussion of illnesses and treatments such as the use of printed materials or Internet, patient self-care, and use of physician assistants or nurse practitioners in primary care which can improve communication and trust [64].

The findings of our study should be interpreted in the context of some limitations. We did not have access to the medical records of the patients. Thus, we utilized participants' responses to survey questions for measuring chronic illnesses. While we recognize the limitations of self-reported measures of mental illness, it should be noted that survey data has been routinely used for surveillance of mental illness and its associated health and cost burden [65]. Also, the MCBS utilizes computer-assisted interview procedures to improve the quality of the survey data. The participants are surveyed at relatively short intervals and the collected information is verified from medical claims to minimize errors [44].

It is possible that patients who reported multiple chronic physical and mental conditions had more critical assessments of their interaction with their provider. Also, communication and trust are container concepts and comprise several different domains [33, 66]. Patients' perceived communication and trust with the doctor might be influenced by several other factors such as the severity of patient's chronic condition, differences in patient and providers' culture and communication styles, and organizational factors [15]. Multimorbidity is, therefore, one of the many factors that affect patient-doctor communication and trust. The study sample included elderly individuals who had at least one chronic physical condition. By excluding patients who have no comorbidities, any differences in communication and trust observed among the three groups were likely due to the presence of multimorbidity only. We did not compare patients with and without chronic conditions as this has been covered in the literature [35]. Further, we did not use a continuous variable for multimorbidity such as number of chronic conditions because we wanted to examine the association of type of multimorbidity with communication and trust. Therefore, we categorized the patients into three groups according to the presence of multiple chronic physical and mental conditions.

The strengths of our study include the use of a comprehensive list of survey items from MCBS AC module to measure specific components of patient-doctor communication and trust, which has been lacking in existing studies. We used a nationally representative dataset with a large sample size, and our study results can be generalized to elderly noninstitutionalized Medicare beneficiaries throughout the US. Also, our study used MCBS, which is a valid multipurpose survey and one of the first to use CAPI for data quality.

## 6. Conclusions

The majority of elderly Medicare beneficiaries experienced effective communication and high trust with their primary care doctors. The elderly with both chronic physical and mental health conditions had ineffective communication with their doctor as compared to those with multiple physical illnesses only. However, multimorbidity did not affect elderly patient's trust in the doctor. Doctor being often in a hurry and doctor often not explaining illnesses were the two most significant communication issues reported by those with multimorbidity. In the absence of evidence-based clinical guidelines, better patient-doctor communication is required to overcome the challenges of managing multiple chronic conditions. It is important to examine and explain the multiple medical conditions and treatments among patients with multimorbidity.

## Figures and Tables

**Table 1:** Description of elderly Medicare beneficiaries by multimorbidity categories. Medicare Current Beneficiary Survey, access to care module 2012.

	Total, wt%	Multimorbidity categories, row wt%			
	*N *= 9,867	No MM	MM-PI	MM-PI&MI	Sig.
All		15.9	72.0	12.1	
*Sociodemographic factors*					
*Sex*					
Female	56.6	13.9	71.6	14.5	*∗∗∗*
Male	43.4	18.4	72.6	9.0	
*Age groups*					
65–69	27.8	20.2	65.5	14.3	*∗∗∗*
70–74	25.8	16.8	71.8	11.5	
75–79	19.1	13.7	75.7	10.6	
≥80	27.3	12.1	76.2	11.7	
*Race/ethnicity*					
Non-Hispanic White	78.5	16.0	71.7	12.2	*∗∗∗*
Non-Hispanic African American	8.0	14.2	76.7	9.1	
Hispanic/Latino	8.7	16.8	66.9	16.3	
Other race	4.7	14.1	76.9	9.1	
*Marital status*					
Married	57.2	17.6	72.0	10.3	*∗∗∗*
Not Married	42.8	13.5	71.9	14.6	
*Education*					
Less than HS	20.6	11.5	73.3	15.2	*∗∗∗*
HS	34.0	15.1	73.0	11.9	
More than HS	45.4	18.3	70.8	10.9	
*Annual personal income*					
<$20,000	27.7	11.1	72.4	16.5	*∗∗∗*
$20,000–$30,000	20.3	15.4	72.1	12.5	
$30,000–$50,000	22.7	16.0	72.8	11.2	
>$50,000	25.2	20.2	71.3	8.5	
Missing	4.1	23.3	68.1	8.5	
*HMO*					
Yes	35.1	16.6	71.5	11.9	
No	64.9	15.5	72.3	12.3	
*Medicaid*					
Yes	12.3	7.6	71.8	20.6	*∗∗∗*
No	87.7	17.0	72.0	11.0	
*Private insurance*					
Yes	54.2	16.6	73.2	10.1	*∗∗∗*
No	45.8	15.1	70.4	14.5	
*Metro status*					
Metro	77.4	16.2	71.6	12.2	
Not Metro	22.6	14.6	73.4	12.0	
*Region*					
Northeast	18.4	17.2	70.9	11.9	*∗*
Midwest	23.4	15.3	73.7	11.0	
South	37.7	14.4	72.8	12.8	
West	20.6	17.9	69.7	12.4	

*Health status*					
*General health status*					
Excellent/very good	50.4	22.9	69.4	7.6	*∗∗∗*
Good	30.0	10.6	77.6	11.8	
Fair/poor	19.6	5.7	70.0	24.4	
*Functional limitations *					
None	66.2	20.3	71.6	8.1	*∗∗∗*
1-2 ADL	23.8	7.9	75.0	17.1	
3–6 ADL	9.9	5.5	67.5	27.0	

*Health condition*					
*Body mass index*					
Underweight/normal	32.4	20.1	69.3	10.6	*∗∗∗*
Overweight	37.0	16.4	71.8	11.8	
Obese/morbid obese	27.6	10.4	75.1	14.5	
Missing	3.0	14.0	75.2	10.8	

*Note.* Based on 9,867 Medicare beneficiaries with age above 65 years and at least one chronic physical health condition. Multimorbidity categories: (a) no MM: no multimorbidity; (b) MM-PI: two or more chronic physical conditions but no mental illness; and (c) MM-MI&PI: both chronic physical and mental conditions. ADL: activities of daily living; HMO: health maintenance organization; HS: high school; Sig.: significance level; wt%: weighted percentage.

^*∗∗∗*^ *p* < .001. ^*∗*^ *p* < .05.

**Table 2:** Weighted percentages of patient-physician communication and trust items: overall and by multimorbidity categories. Medicare Current Beneficiary Survey, access to care module 2012.

Survey items	Strongly agree	Agree	Disagree	Strongly disagree	No MM	MM-PI	MM-PI&MI	Sig.
	Weighted percentages				^a^Column weighted percentages			
*Patient-physician communication*								
(1) Your doctor tells you all you want to know about your condition and treatment.	38.9	56.8	3.9	0.4	97.5	95.8	93.3	*∗∗∗*
(2) Your doctor often does not explain your medical problems to you.	1.5	6.3	65.6	26.6	6.5	7.5	11.0	*∗∗∗*
(3) You often have health problems that should be discussed but are not.	0.5	5.9	68.1	25.5	4.1	6.1	11.6	*∗∗∗*
(4) Your doctor often seems to be in a hurry.	2.1	10.4	62.6	24.9	8.0	13.0	15.4	*∗∗∗*
(5) Your doctor often acts as though he was doing you a favor by talking to you.	0.5	3.5	59.2	36.8	3.1	3.8	5.6	*∗∗*
(6) Your doctor answers all questions.	41.4	56.1	2.2	0.3	98.4	97.7	95.5	*∗∗∗*
*Patient-physician trust*								
(1) Your doctor is very careful to check everything when examining you.	46.7	48.7	4.2	0.4	97.4	95.4	92.9	*∗∗∗*
(2) Your doctor is competent and well-trained.	51.6	47.5	0.7	0.2	99.3	99.1	99.0	
(3) Your doctor has a good understanding of your medical history.	49.4	48.3	2.0	0.3	97.9	97.8	96.9	
(4) Your doctor has a complete understanding of the things that are wrong with you.	45.1	51.5	3.1	0.3	97.2	96.8	94.8	*∗∗*
(5) You have great confidence in your doctor.	46.7	49.9	2.9	0.5	97.8	96.7	94.8	*∗∗*

*Note.* ^a^Column weighted percentages of beneficiaries by multimorbidity categories who agreed or strongly agreed with the survey item.

Multimorbidity categories: (a) no MM: no multimorbidity; (b) MM-PI: two or more chronic physical conditions but no mental illness; and (c) MM-MI&PI: both chronic physical and mental conditions.

Sig.: significance level from chi-square tests.

^*∗∗∗*^ *p* < .001. ^*∗∗*^ *p* < .01.

**Table 3:** Description of elderly Medicare beneficiaries by patient-doctor communication and trust. Medicare Current Beneficiary Survey, access to care module 2012.

	Effective communication		High trust	
All	*N* = 8,298		*N* = 5,102	
	Row wt%	Sig.	Row wt%	Sig.
*Multimorbidity*				
No MM	89.7	*∗∗∗*	53.7	
MM-PI	84.2		53.2	
MM-PI&MI	80.1		52.2	

*Sociodemographic factors*				
*Sex*				
Women	84.3		53.3	
Men	84.9		53.0	
*Age in years *				
65–69 yrs.	85.6		56.2	*∗∗∗*
70–74 yrs.	85.2		55.0	
75–79 yrs.	84.1		52.4	
>80 yrs.	83.3		48.8	
*Race/ethnicity*				
Non-Hispanic White	85.2	*∗*	55.0	*∗∗∗*
Non-Hispanic African American	82.2		43.2	
Hispanic/Latino	83.3		48.8	
Other race^†^	80.8		46.6	
*Marital status*				
Married	85.6	*∗∗*	55.2	*∗∗∗*
Not married^*α*^	83.2		50.3	
*Education*				
Less than high school	80.2	*∗∗∗*	43.2	*∗∗∗*
High school	84.2		50.2	
Above high school	86.8		60.0	
*Annual personal income*				
<$20,000	81.7	*∗∗∗*	45.8	*∗∗∗*
$20,000–$30,000	83.1		50.4	
$30,000–$50,000	85.7		54.7	
>$50,000	87.8		62.6	
Missing	85.1		49.7	
*HMO enrollment*				
Yes	84.4		52.6	
No	84.7		53.5	
*Medicaid insurance*				
Yes	80.6	*∗∗∗*	45.7	*∗∗∗*
No	85.1		54.2	
*Private insurance*				
Yes	85.5	*∗∗*	55.2	*∗∗∗*
No	83.4		50.7	
*Metro status*				
Metro	85.1	*∗*	55.2	*∗∗∗*
Not metro	82.6		46.0	
*Geographic region*				
Northeast	86.2		54.5	
Midwest	86.0		55.6	
South	83.4		50.3	
West	83.6		54.5	

*Health status*				
*General health*				
Excellent/very good	87.7	*∗∗∗*	59.4	*∗∗∗*
Good	83.3		47.8	
Fair/poor	78.6		45.4	
*Functional limitations*				
None	86.5	*∗∗∗*	55.3	*∗∗∗*
1-2 ADL	81.5		48.9	
3–6 ADL	79.2		49.2	

*Health condition*				
*Body mass index*				
Underweight/normal	84.9		53.3	
Overweight	85.3		53.8	
Obese/morbid obese	83.2		52.5	
Missing	84.6		49.8	

*Satisfaction with care*				
*Satisfaction with care*				
Yes	85.2	*∗∗∗*	53.7	*∗∗∗*
No	60.6		36.2	

*Note.* Based on 9,867 Medicare beneficiaries with age above 65 years and at least one chronic physical health condition. Multimorbidity categories: (a) no MM: no multimorbidity; (b) MM-PI: two or more chronic physical conditions but no mental illness; and (c) MM-MI&PI: both chronic physical and mental conditions.

^†^Asian, Native Hawaiian or Pacific Islander, American Indian or Alaska Native, more than one, or others. ^*α*^Widowed/divorced/separated/never married. ADL: activities of daily living; HMO: health maintenance organization; Sig.: significance level from chi-square tests; wt%: weighted percentage.

^*∗∗∗*^ *p* < .001. ^*∗∗*^ *p* < .01. ^*∗*^ *p* < .05.

**Table 4:** Adjusted Odds Ratios and 95% Confidence Intervals for patient-doctor communication and trust from logistic regression models. Medicare Current Beneficiary Survey, access to care module 2012.

	Effective communication			High trust		
	AOR	95% CI	Sig.	AOR	95% CI	Sig.
*Multimorbidity*						
No MM	1.48	[1.21, 1.82]	*∗∗∗*	0.95	[0.84, 1.07]	
MM-PI&MI	0.80	[0.68, 0.96]	*∗*	0.98	[0.84, 1.15]	
MM-PI	Ref.					

*Sociodemographic factors*						
*Sex*						
Female	1.06	[0.94, 1.18]		1.15	[1.04, 1.28]	*∗∗*
Male	Ref.					
*Age in years *						
70-74	0.98	[0.81, 1.17]		0.97	[0.83, 1.12]	
75–79	0.94	[0.79, 1.12]		0.94	[0.82, 1.07]	
>80	0.90	[0.76, 1.06]		0.82	[0.71, 0.94]	*∗∗*
65–69	Ref.					
*Race/ethnicity*						
African American	0.93	[0.74, 1.17]		0.71	[0.59, 0.84]	*∗∗∗*
Latino	1.01	[0.79, 1.27]		0.88	[0.72, 1.07]	
Other race^†^	0.82	[0.62, 1.07]		0.76	[0.61, 0.95]	*∗*
White	Ref.					
*Marital status*						
Married	1.05	[0.90, 1.22]		1.01	[0.91, 1.12]	
Widowed/divorced/separated/never married	Ref.					
*Education status*						
High school	1.17	[1.00, 1.36]	*∗*	1.14	[1.02, 1.28]	*∗*
≥College	1.35	[1.11, 1.63]	*∗∗*	1.53	[1.32, 1.77]	*∗∗∗*
<High school	Ref.					
*Annual personal income *						
$20,000–$30,000	0.97	[0.80, 1.17]		1.10	[0.96, 1.27]	
$30,000–$50,000	1.12	[0.90, 1.39]		1.22	[1.05, 1.42]	*∗∗*
>$50,000	1.22	[0.96, 1.54]		1.02	[0.79, 1.32]	
<$20,000	Ref.					
*HMO enrollment *						
Yes	0.99	[0.84, 1.17]		0.96	[0.85, 1.08]	
No	Ref.					
*Medicaid insurance *						
Yes	1.00	[0.81, 1.24]		0.95	[0.79, 1.14]	
No	Ref.					
*Private insurance *						
Yes	1.01	[0.87, 1.17]		1.04	[0.92, 1.17]	
No	Ref.					
*Metro status*						
Yes	0.89	[0.74, 1.06]		0.73	[0.60, 0.88]	*∗∗∗*
No	Ref.					
*Region*						
Midwest	1.01	[0.78, 1.31]		1.10	[0.92, 1.31]	
South	0.83	[0.66, 1.06]		0.89	[0.74, 1.07]	
West	0.77	[0.61, 0.97]	*∗*	0.92	[0.73, 1.16]	
Northeast	Ref.					

*Health condition*						
*Body mass index *						
Overweight	1.04	[0.91, 1.19]		1.03	[0.92, 1.16]	
Obese/morbid obese	0.92	[0.78, 1.08]		0.99	[0.89, 1.12]	
Underweight/normal	Ref.					

*Satisfaction with care*						
*Satisfaction with care*						
Yes	3.38	[2.63, 4.36]	*∗∗∗*	1.82	[1.35, 2.47]	*∗∗∗*
No	Ref.					

*Note*. Based on 9,867 Medicare beneficiaries with age above 65 years and at least one chronic physical health condition. Multimorbidity categories: (a) no MM: no multimorbidity; (b) MM-PI: two or more chronic physical conditions but no mental illness; and (c) MM-MI&PI: both chronic physical and mental conditions.

^†^Asian, Native Hawaiian or Pacific Islander, American Indian or Alaska Native, more than one, or others.

AOR: Adjusted Odds Ratio; CI: Confidence Interval; HMO: Health Maintenance Organization; Ref.: reference; Sig.: significance level from logistic regression model.

^*∗∗∗*^ *p* < .001. ^*∗∗*^ *p* < .01. ^*∗*^ *p* < .05.
